# Gam-COVID-Vac, EpiVacCorona, and CoviVac effectiveness against lung injury during Delta and Omicron variant surges in St. Petersburg, Russia: a test-negative case–control study

**DOI:** 10.1186/s12931-022-02206-3

**Published:** 2022-10-10

**Authors:** Anton Barchuk, Anna Bulina, Mikhail Cherkashin, Natalia Berezina, Tatyana Rakova, Darya Kuplevatskaya, Dmitriy Skougarevskiy, Artemiy Okhotin

**Affiliations:** 1 Institute for Interdisciplinary Health Research, European University at St. Petersburg, Shpalernaya Ulitsa 1, 191187 St. Petersburg, Russia; 2 Medical Institute Named After Berezin Sergey, Esenina Ulitsa 2-3a, 194354 St. Petersburg, Russia; 3 Tarusa Hospital, Karla Libknekhta Ulitsa 16, 249100 Tarusa, Russia

**Keywords:** Vaccine effectiveness, Case–control study, SARS-CoV-2, COVID-19, Lung injury

## Abstract

**Background:**

Monitoring vaccine effectiveness (VE) remains a priority for epidemiological research throughout the COVID-19 pandemic. VE against infection declines with the emergence of new SARS-CoV-2 variants of concern (VOC), but VE against the severe disease remains high. Therefore, we aimed to estimate the effectiveness of COVID-19 vaccines used in Russia against lung injury during Delta and Omicron VOC surges.

**Methods:**

We designed a case–control study (test-negative design) to estimate VE against any (any volume of involved lung parenchyma) and severe (>50% of involved parenchyma) lung injury detected on computer tomography and associated with COVID-19 between October 1, 2021–April 28, 2022 (Delta VOC dominance period followed by Omicron dominance period). We included the data of patients with symptomatic confirmed SARS-CoV-2 infection referred to the low-dose computer tomography triage centres.

**Results:**

Among 23996 patients in the primary analysis, 13372 (55.7%) had any lung injury, and 338 (1.4%) had severe lung injury. The adjusted for age, sex and triage centre VE estimates against any lung injury were 56% (95% confidence interval 54–59) for two-dose Gam-COVID-Vac (Sputnik V), 71% (68–74) for three-dose Gam-COVID-Vac (booster), 2% (−27 to 24) for EpiVacCorona, and 46% (37–53) for CoviVac. VE estimates against severe lung injury were 76% (67–82) for two-dose Gam-COVID-Vac (Sputnik V), 87% (76–93) for three-dose Gam-COVID-Vac, 36% (−63 to 75) for EpiVacCorona, and 80% (45–92) for CoviVac.

**Conclusions:**

Gam-COVID-Vac remained effective against lung injury associated with COVID-19 during Delta and Omicron VOC surges, and one Gam-COVID-Vac booster could be seen as an appropriate option after a two-dose regimen. CoviVac was also effective against lung injury. EpiVacCorona use in population-based vaccination should be halted until effectiveness and efficacy evidence is provided.

*Trial registration* The joint study of COVID-19 vaccine effectiveness in St. Petersburg was registered at ClinicalTrials.gov (NCT04981405, date of registration—August 4, 2021).

**Supplementary Information:**

The online version contains supplementary material available at 10.1186/s12931-022-02206-3.

## Background

Monitoring vaccine effectiveness (VE) remains a priority for epidemiological research throughout the COVID-19 pandemic caused by severe acute respiratory syndrome coronavirus (SARS-CoV-2) [1]. Several studies showed that VE declines with the emergence of new SARS-CoV-2 variants of concern [2–4]. However, the protection against the severe disease associated with COVID-19 remains high for globally used vaccines [5, 6]. Still, the protection levels could vary worldwide [7]. Three vaccines are used in Russia for the population-based immunisation against SARS-CoV-2. Initial Gam-COVID-Vac (Sputnik V) vaccine efficacy study results raised some concerns [8]. However, several subsequent effectiveness reports suggested that Gam-COVID-Vac remained highly effective during subsequent SARS-CoV-2 surges, including Delta variant of concern (VOC) [5, 9]. The most recent report from St. Petersburg showed that in contrast to Gam-COVID-Vac, two other vaccines used in Russia, EpiVacCorona and CoviVac, were not similarly effective against symptomatic infection during the SARS-CoV-2 Delta VOC surge [10]. However, both vaccines were relatively rarely used compared to Gam-COVID-Vac, and the study was underpowered to detect their effectiveness. Unfortunately, efficacy data are available only for Gam-COVID-Vac [11] and not from EpiVacCorona and CoviVac manufacturers.

On January 10, 2022, the number of newly registered SARS-CoV-2 cases started to rapidly rise in St. Petersburg, Russia, marking the start of the Omicron VOC surge. A report from South Africa suggests significantly reduced odds of hospitalisation among individuals with Omicron variant infection [12]. However, inferring disease severity in the settings of increasing natural and vaccine immunity is challenging. Reduced severity may be partly accounted for protection due to vaccination. Despite multiple studies, data on vaccine effectiveness for all vaccines during the Omicron variant surge are still lacking, including reports for globally used Gam-COVID-Vac.

Among studies that address the VE during new SARS-CoV-2 VOC surges, case–control, and more particular, test-negative design studies, play a crucial role as they provide a rapid assessment for VE [13]. This study design is subject to several biases, but it became a robust epidemiological tool used in different settings. More often, a test-negative design was used to assess VE against infection [4, 14–16]. However, it can also be applied to determine effectiveness against severe outcomes associated with COVID-19. A previous study in St. Petersburg used information on low-dose computed tomography triage to assess VE against severe lung injury associated with COVID-19 and subsequent referral to the hospital [9]. In this population-based case-control study (test-negative study design), we aimed to estimate the effectiveness of the Russian COVID-19 vaccines against lung injury associated with COVID-19 during the SARS-CoV-2 Delta and Omicron variant surge in St. Petersburg, Russia, between October 2021 and April 2022.

## Methods

### Settings, population and study design

In this study, we included data on individuals referred to two outpatient centres of the Medical Institute named after Berezin Sergey (MIBS), a private medical facility contracted by the city government to provide low-dose computed tomography (LDCT) triage service. The St. Petersburg city government offered this free service during the COVID-19 pandemic to all citizens of the city who experienced symptoms and had confirmed or suspected SARS-CoV-2 infection. This setting has been previously used to assess VE against referral to the hospitalisation in August 2021 and against symptomatic SARS-CoV-2 infection in October 2021 [9, 10].

Patients referred to LDCT had either a positive polymerase chain reaction (PCR) test for SARS-CoV-2, or their test results were not available, pending and even negative (especially in the later period when more patients with persistent respiratory symptoms were referred to triage). Therefore, we excluded patients with non-positive or equivocal SARS-CoV-2 PCR test results (e.g. patients with two tests with different results) and a history of confirmed SARS-CoV-2 infection from our primary analysis. However, we have included all patients in our sensitivity analysis.

We retrospectively collected individual-level data from outpatient triage centres related to patients referred to the LDCT triage between October 1, 2021, and April 28, 2022, during the Delta and the Omicron VOC surges in St. Petersburg. We did not perform any additional attempts to actively recruit patients to LDCT triage. All patients referred to LDCT triage underwent brief physical examination, including pulse oximetry. We used this setting to design a case–control study with “other patient” controls (test-negative study) [17] to determine the VE against any lung injury in symptomatic patients with SARS-CoV-2 infection. Test-negative design was broadly used to assess VE against SARS-CoV-2 infection detected using laboratory tests, but we applied it to assess VE against lung injury seen on LDCT. A similar design with imaging workup was previously used to determine the risk of venous thrombosis [18]. Cases were patients with a lung injury detected on LDCT (objective diagnosis of COVID-19 pneumonia). Those referred with SARS-CoV-2 infection but who had no lung injury served as control subjects. When planning our study, we followed the WHO interim guidance to evaluate COVID-19 vaccine effectiveness [19].

### Vaccination status

Vaccination status was self-reported. Patients referred to LDCT triage were asked about vaccine type, the number of doses, and dates for the doses. Three vaccines were approved for primary vaccination during the pandemic in Russia: Gam-COVID-Vac [11] two-dose (Sputnik V) and one-dose regimen (Sputnik Light), EpiVacCorona [20], and CoviVac [21] (both two-dose regimens). Gam-COVID-Vac is an adenovirus viral vector vaccine that comes in two doses 21 days apart. Doses are based on two human adenoviruses: Ad26 (serotype 26) and Ad5 (serotype 5). The viruses contain the gene that encodes the full-length spike protein (S) of SARS-CoV-2. Gam-COVID-Vac one-dose (Sputnik Light based on Ad26) was also recommended as the preferred option for the booster after COVID-19 infection. EpiVacCorona is a peptide-based vaccine. EpiVacCorona includes three peptides of the spike protein and a chimeric protein (two parts) with the polyhistidine-tag. EpiVacCorona is recommended in two doses, 14–21 days apart. CoviVac is an inactivated virus-based vaccine based on an inactivated SARS-CoV-2 strain AYDAR-1 and mixed with an aluminium-based adjuvant. CoviVac is also recommended in two doses, 14 days apart.

Vaccine status was based on the number of doses, date of the last dose, and vaccine type. Participants who reported two doses of Gam-COVID-Vac, EpiVacCorona, and CoviVac and received the last dose at least 14 days before the referral to LDCT were considered fully vaccinated with corresponding vaccines. For EpiVacCorona and CoviVac, participants who did not satisfy criteria for full vaccination status, but reported at least one dose 14 days before the referral were considered partially vaccinated. In the case of Gam-COVID-Vac, participants who reported one dose of Gam-COVID-Vac or Sputnik Light at least 14 days before the referral were considered vaccinated with Sputnik Light. Participants who reported both Gam-COVID-Vac two-dose and booster with Sputnik Light at least 14 days before referral were considered to have three doses or booster with Sputnik Light after Sputnik V. All other combinations of vaccine regimens were related to the “other vaccines” group. Participants who did not report vaccination or did not satisfy the criteria for 14 days after the first dose were considered unvaccinated.

### Outcomes

A local computed tomography score (CT-score) with five gradations (0, 1, 2, 3, 4) which are related to the volume of involved lung parenchyma or percentage of involved lung segments (0, < 25%, 25–50%, 50–75%, 75–100%) was implemented in Russia to assess the severity of the COVID-19 lung injury [22]. The primary outcome was any lung injury reported by LDCT in the triage centre (CT-score 1, 2, 3, or 4). Cases were patients with any lung injury detected on LDCT, and those who had no lung injury served as control subjects.

The secondary outcome was severe lung injury, defined as>50% lung involvement (CT-score 3 or 4). Cases were patients with > 50% lung injury detected on LDCT, and those who had no lung injury served as control subjects.

We did not use the hospital referral as an outcome in contrast to our previous study [9] because the official criteria for hospitalisation changed in the autumn of 2021 in St. Petersburg, forcing older patients to be referred to the hospital regardless of the severity of lung injury.

### Statistical analysis

We used unconditional logistic regression for our primary and secondary outcomes to estimate odds ratios (ORs) for vaccination status among cases and controls, which approximates ORs for the outcomes (any lung injury and > 50% lung injury) among the vaccinated and non-vaccinated patients. The VE was calculated as $100\%\times (1-OR)$ adjusted for age (continuous variable), sex, and the triage LDCT centre. In the additional analysis, we split our database into two periods representing Delta and Omicron VOC surges (October 1, 2021–January 9, 2022, and January 10, 2022–April 28, 2022). We also calculated VE in different age groups adjusting for gender and the triage LDCT centre, and VE for men and women, adjusting for age and the triage LDCT centre. Cases with missing data were excluded from the analyses.

We used R version 4.1.1 (2021-08-10) and R Studio version 2021.9.0.351 for our analyses. Unconditional logistic regression models (without and with variables used for adjustment) were fitted to obtain the odds ratio by taking the exponential of corresponding coefficients. We included age, sex and centre variables in the model for the adjusted analysis. All standard errors and confidence intervals were adjusted for heteroskedasticity with the Huber-Eicker-White sandwich estimator. All estimates are reported with 95% confidence intervals. We abstained from post-hoc sample size calculations [23].

## Results

**Fig. 1:**
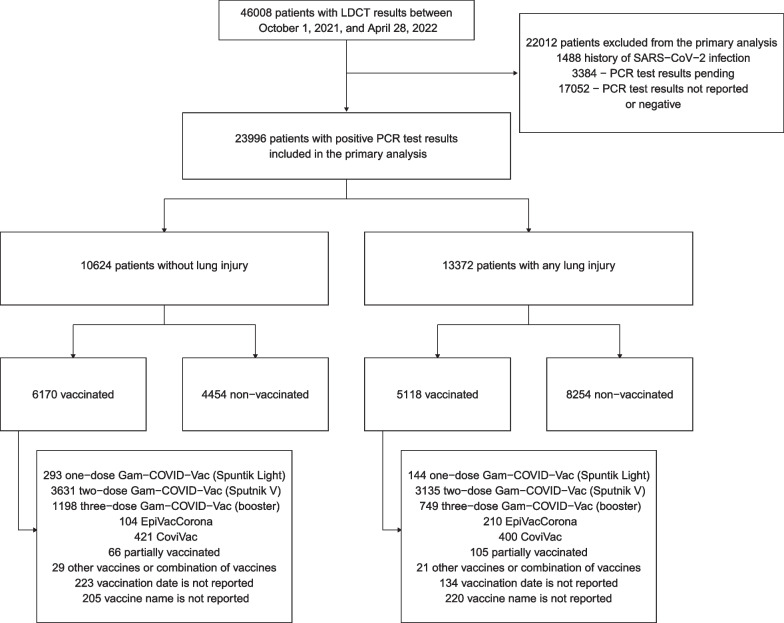
Study flowchart based on the primary outcome

**Fig. 2:**
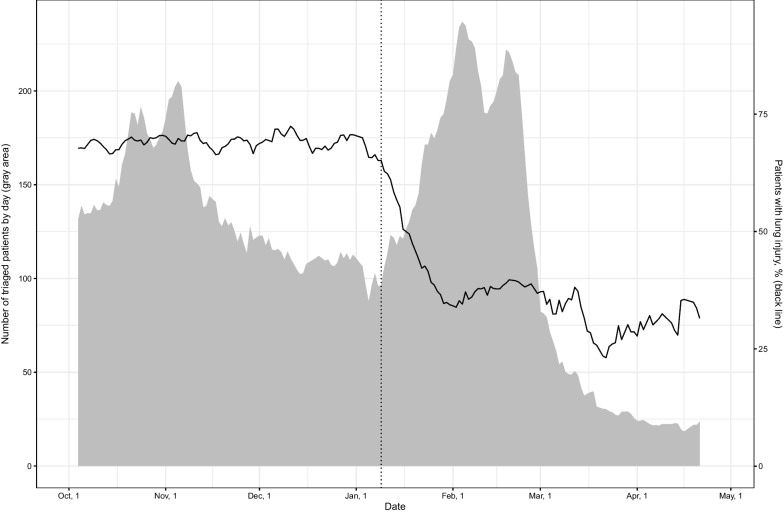
Patients dynamics and proportion of patients with any lung injury through the study period October 2021–April 2022 (dashed vertical line marks the start of the Omicron surge)

Overall, 46008 patients referred to the LDCT triage centres were extracted for the analysis. We included 23996 patients who had SARS-CoV-2 infection confirmed by PCR test in the primary analysis, and 22012 patients were excluded, and for the majority of them, the PCR test result was negative or not reported. Patient characteristics are presented in Table 1, and our study flowchart is presented in Fig. 1. We performed a sensitivity analysis using data from all 46008 patients (Additional file 1: Table A1). The peak number of patients referred to the LDCT triage was observed in January–March 2022, followed by October–November 2021. In contrast to the number of patients referred, the proportion of patients with lung injury has dropped starting from January 2022 (Fig. 2).

**Table 1:** Characteristics of patients with positive PCR test results referred to the LDCT Triage

		Overall	No lung injury	Any lung injury	Severe lung injury
		23996	10624	13372	338
Age mean (SD)		51.62 (16.46)	47.01 (16.27)	55.28 (15.68)	61.70 (15.17)
Age	18–30	2572 (10.7)	1801 (17.0)	771 (5.8)	6 (1.8)
categories (%)	31-40	4490 (18.7)	2484 (23.4)	2006 (15.0)	26 (7.7)
	41–50	4437 (18.5)	2075 (19.5)	2362 (17.7)	53 (15.7)
	51–60	4664 (19.4)	1816 (17.1)	2848 (21.3)	67 (19.8)
	60+	7833 (32.6)	2448 (23.0)	5385 (40.3)	186 (55.0)
Sex (%)	Female	15577 (64.9)	6974 (65.6)	8603 (64.3)	190 (56.2)
	Male	8419 (35.1)	3650 (34.4)	4769 (35.7)	148 (43.8)
Vaccination	Non-vaccinated	12708 (53.0)	4454 (41.9)	8254 (61.7)	257 (76.0)
status	One-dose Gam-COVID-Vac	437 (1.8)	293 (2.8)	144 (1.1)	1 (0.3)
	Two-dose Gam-COVID-Vac	6766 (28.2)	3631 (34.2)	3135 (23.4)	52 (15.4)
	Three-dose Gam-COVID-Vac	1947 (8.1)	1198 (11.3)	749 (5.6)	11 (3.3)
	EpiVacCorona	314 (1.3)	104 (1.0)	210 (1.6)	5 (1.5)
	CoviVac	821 (3.4)	421 (4.0)	400 (3.0)	4 (1.2)
	No vaccination date	357 (1.5)	223 (2.1)	134 (1.0)	2 (0.6)
	Vaccine name is not reported	425 (1.8)	205 (1.9)	220 (1.6)	2 (0.6)
	Partially vaccinated	171 (0.7)	66 (0.6)	105 (0.8)	4 (1.2)
	Other vaccines	50 (0.2)	29 (0.3)	21 (0.2)	0 (0.0)
Period (%)	Delta surge				
	(Oct 1, 2021–Jan 9, 2022)	13394 (55.8)	4141 (39.0)	9253 (69.2)	244 (72.2)
	Omicron surge				
	(Jan 10, 2022–Apr 28, 2022)	10602 (44.2)	6483 (61.0)	4119 (30.8)	94 (27.8)
Triage	1	15689 (65.4)	7266 (68.4)	8423 (63.0)	210 (62.1)
Centre (%)	2	8307 (34.6)	3358 (31.6)	4949 (37.0)	128 (37.9)

Among all patients included in the primary analysis, 11,288 (47%) were vaccinated (at least one dose 14 days before the referral): 437 (1.8%) received one dose of Gam-COVID-Vac (Sputnik Light), 6766 (28.2%) received two doses of Gam-COVID-Vac (Sputnik V), 1947 (8.1%)—one dose of Gam-COVID-Vac booster after two doses Gam-COVID-Vac, 314 (1.3%) received EpiVacCorona, and 821 (3.4%)—CoviVac. Additionally, 1003 (4.2%) were partially vaccinated, used other vaccines or vaccine regimens, or the vaccine name or vaccination date was not reported. Among 23,996 patients in the primary analysis, 13,372 (55.7%) had any lung injury, and 338 (1.4%) had severe lung injury.

In the primary analysis, the adjusted (for age, sex and the triage LDCT centre) VE against any lung injury was 74% (95% confidence interval 58–79) for one-dose Gam-COVID-Vac (Sputnik Light), 56% (54–59) for two-dose Gam-COVID-Vac (Sputnik V), 71% (68–74) for three-dose Gam-COVID-Vac (booster), 2% (−27 to 24) for EpiVacCorona and 46% (37–53) for CoviVac. VE against severe lung injury was higher for all vaccines in our study (Table 2). VE estimates were slightly lower in women except for CoviVac (Table 3). The VE estimates were slightly lower during the Omicron surge (January 10, 2022–April 28, 2022) compared to the Delta surge (January 10, 2022–April 28, 2022) except for three-dose Gam-COVID-Vac and EpiVacCorona. However, in both study periods, confidence intervals for the VE of EpiVacCorona include zero (Table 3).

**Table 2:** Effectiveness of vaccination against any and severe lung injury

	VE against any lung injury		Severe lung injury	
	Crude (95% confidence interval)	Adjusted for age, sex and triage center (95% confidence interval)	Crude (95% confidence interval)	Adjusted for age, sex and triage center (95% confidence interval)
One-dose Gam-COVID-Vac	73% (68 to 78)	74% (68 to 79)	94% (58 to 99)	94% (60 to 99)
Two-dose Gam-COVID-Vac	53% (51 to 56)	56% (54 to 59)	75% (66 to 82)	76% (67 to 82)
Three-dose Gam-COVID-Vac	66% (63 to 69)	71% (68 to 74)	84% (71 to 91)	87% (76 to 93)
EpiVacCorona	− 9% (− 38 to 14)	2% (− 27 to 24)	17% (− 106 to 66)	36% (− 63 to 75)
CoviVac	49% (41 to 56)	46% (37 to 53)	84% (56 to 94)	80% (45 to 92)

**Table 3:** Effectiveness of vaccination against any lung injury, according to age group, sex and study period

		One-dose Gam-COVID-Vac (Sputnik Light)	Two-dose Gam-COVID-Vac (Sputnik V)	Three-dose Gam-COVID-Vac (booster)	EpiVacCorona	CoviVac
Age (categories)	18–30	81% (50 to 92)	67% (58 to 73)	74% (60 to 83)	-60% (− 309 to 37)	28% (− 13 to 55)
	31–40	69% (48 to 82)	67% (61 to 71)	68% (58 to 76)	− 8% (− 97 to 41)	63% (48 to 74)
	41–50	70% (53 to 81)	56% (49 to 61)	65% (56 to 72)	− 36% (− 137 to 22)	34% (12 to 51)
	51–60	76% (64 to 84)	55% (48 to 61)	69% (61 to 75)	0% (− 72 to 42)	44% (25 to 57)
	61 +	78% (68 to 84)	50% (43 to 55)	74% (70 to 78)	33% (0 to 55)	57% (40 to 69)
Sex	Female	70% (62 to 77)	56% (53 to 60)	71% (67 to 75)	− 11% (− 53 to 20)	49% (39 to 57)
	Male	81% (72 to 87)	57% (52 to 61)	71% (65 to 76)	20% (− 20 to 47)	37% (19 to 52)
Period	Delta	57% (32 to 72)	59% (55 to 62)	57% (48 to 64)	− 35% (− 100 to 9)	41% (27 to 52)
	Omicron	54% (40 to 64)	38% (32 to 44)	57% (51 to 63)	5% (− 43 to 37)	30% (12 to 44)

The sensitivity analysis results (performed using data from 46,008 patients referred to the LDCT triage) were not fundamentally different from the primary analysis results (Additional file 1: Table A2).

## Discussion

This is the first study examining the effectiveness of different vaccine regimens used in Russia against lung injury associated with COVID-19 during both Omicron and Delta surges. Our study showed that Gam-COVID-Vac (Sputnik V and Sputnik Light), the most widely-used vaccine in Russia, remained effective against lung injury associated with COVID-19 through the Delta VOC surge in October–December 2021 and was also effective during Omicron VOC surge in January–April 2022. In addition, the Gam-COVID-Vac VE against any lung injury of 56% was similar to our previous study results that focused on the effectiveness of vaccination on severe lung injury and hospitalisation during the Delta VOC surge in St. Petersburg in July and August 2021 [9]. Our results supplement the available evidence on Gam-COVID-Vac [5] and provide the first data from the Omicron surge, supporting similar studies of other vaccines [24]. VE for most vaccines dropped slightly during the Omicron surge.

Our study also provides the first assessments of Gam-COVID-Vac two-dose vaccination followed by Gam-COVID-Vac booster. After two-dose vaccination, participants who reported additional Gam-COVID-Vac booster had VE against any lung injury equal to 71%. These results are in line with data obtained for other vaccines [25, 26]. The effectiveness of one-dose Gam-COVID-Vac was 74%, but it is likely that these results reflect the effectiveness of the post-infection booster, rather than VE per se.

The only Russian vaccine that failed to show effectiveness against lung injury in our study was EpiVacCorona. This supports the study results that assessed EpiVacCorona against symptomatic SARS-CoV-2 infection in St. Petersburg [9]. While the previous study was underpowered, this study provides better information on the lack of VE for EpiVacCorona despite the low vaccine uptake in Russia. We believe these studies’ results have direct policy implications. EpiVacCorona in a population-based vaccination programme should be discouraged, especially during the new SARS-CoV-2 variant surges.

CoviVac is another vaccine used almost exclusively in Russia. Unfortunately, population-based vaccination with CoviVac was started before any efficacy results were available. And unlike Gam-COVID-Vac, the CoviVac Phase III trial is still underway without final results yet. Our study results show that CoviVac VE against any lung injury equals 46%. CoviVac is an inactivated virus-based COVID-19 vaccine, and a similar comparator could be the Sinovac or Sinopharm BIBP COVID-19 vaccine [27, 28]. We did not perform a direct comparative assessment of the vaccines in our study. Still, studies on VE in the countries where several vaccines, including Sinovac, were used showed similar results. In the study in Hungary, Sinovac was effective against SARS-CoV-2 infection and COVID-19-associated deaths, but its effectiveness against infection was lower than for vector-based and mRNA vaccines [5]. Despite such a delay in the efficacy study results publication, the CoviVac Phase III study is still highly anticipated. The evolution of SARS-CoV-2 may require a combined vaccine regimen. In addition, our results suggest that CoviVac remained effective during the Omicron surge, in contrast to laboratory evidence of sub-optimal neutralisation of the Omicron variant for inactivated vaccines [29]. These results highlight the importance of more laboratory and epidemiological data for various vaccines available worldwide, as making conclusions based only on the type of the vaccines is not enough to infer effectiveness [30].

Another study finding was related to lung injury prevalence through the waves of the pandemic in St. Petersburg. Lung injury was significantly less common in the Omicron surge than in the previous Delta wave. It supports the studies that showed disease severity during Omicron surge to be lower than during previous peaks [12, 31]. This difference can only be partly accounted for by the increase in natural immunity in St. Petersburg. At the same time, the VE of Gam-COVID-Vac booster against lung injury was similar during the Delta and Omicron surges, supporting other pieces of evidence that vaccination is an effective tool against severe disease.

The major limitation of the study is the self-reported status of vaccination. Participants were asked about the type of vaccine, number of doses and dates and we assume some level of misclassification due to the self-reported nature of this information. For example, we have participants who reported only the date (1.8%) or only the name of the vaccine (1.5%). We did not include these patients in particular vaccine groups. We do not think this bias was differential and could dramatically bias our results, but it should be considered when assessing our study results.

Several limitations of our study are inherent in the observational study design. First, selection could have happened due to the health-seeking behaviour of study participants. This bias could impact the results if healthier vaccinated participants were more likely to be referred to LDCT. Second, we could not exclude the scenario when vaccinated individuals avoided additional diagnostic work-up in the presence of illness. Unfortunately, our data do not allow us to assess the direction of this bias.

We excluded patients who reported positive PCR in the past (before the index episode), but we had only 1488 patients with this information. However, many individuals had asymptomatic or mild COVID-19 without PCR confirmation. The VE could also represent the combined effect of vaccines and past COVID-19, especially for one-dose Gam-COVID-Vac, which was a recommended option for individuals with a history of SARS-CoV-2 infection. It is essential to mention that we estimated only lung-related COVID-19 complications while we could miss important extrapulmonary COVID-19 presentations.

The major strength of our study is the objective nature of our outcome. The information reported after LDCT is available for all included patients, and it was assessed consistently through the course of the pandemic in triage centres. The classification for lung injury was implemented in the early pandemic [22], and we are not aware of any changes in the application of this classification. Computed tomography was used as an additional COVID-19 diagnostic tool during this pandemic, but our study takes advantage of these data in applying a test-negative design to assess VE. We haven’t found any other research with a similar design and outcome. Still, it would be important to determine the robustness of our study results by applying a similar study design in different settings and for various vaccines.

Based on data from triage centres, we established a pre-existing framework for the real-time assessment of VE through the pandemic. In addition, we expanded the information we collected from the simple vaccination status to particular vaccine types starting from October 2021. This independently established framework was effectively used to monitor the VE in the absence of other effectiveness studies in Russia. There are opportunities for conducting high-quality basic epidemiological studies in Russia, but barriers to implementing their results in public health decision-making need to be studied. Though this issue probably is beyond the scope of our study.

EpiVacCorona population-based vaccination should be halted unless efficacy and effectiveness study results are available. It was a systematic flaw to start population-based vaccination in Russia before phase III trial results for any vaccine were available. However, our study is the only known real-world evidence of EpiVacCorona, suggesting that it was ineffective against lung injury associated with COVID-19 during Delta and Omicron VOC surges.

In conclusion, Gam-COVID-Vac remains effective against lung injury associated with COVID-19 caused by new variants of SARS-CoV-2, and a Gam-COVID-Vac booster can be seen as an appropriate option after a two-dose regimen. Estimating effectiveness remains a challenge due to the high prevalence of natural immunity in the population and changing properties of the infectious agent. EpiVacCorona was not effective against any lung injury during Delta and Omicron VOC surges based on our study results. Therefore, EpiVacCorona use in population-based vaccination should be halted until additional effectiveness and efficacy evidence is provided. Despite optimistic results obtained in our study, CoviVac efficacy and safety data are still required to justify its use in a population-based vaccination. More independent studies are needed to monitor the effectiveness of vaccines in the later phases of the COVID-19 pandemic as it provides additional insight into the future of global SARS-CoV-2 impact.

## Supplementary information

**Additional file 1: Table A1**. Characteristics of all patients referred to the LDCT triage. **Table A2**. Effectiveness of vaccination against any and severe lung injury (sensitivity analysis performed using data from 46008patients referred to the LDCT triage.

## Data Availability

All analyses were conducted in R [32], study data and code is available online https://github.com/eusporg/spb_covid_study20.

## Declarations

### Patient and public involvement

No patients were involved in setting the research question or the outcome measures, nor were they involved in developing plans for the design or implementation of the study. No patients were asked to advise on the conduct, reporting, or dissemination plans of our research.
