# Supplementary material for: Gam-COVID-Vac, EpiVacCorona, and CoviVac effectiveness against lung injury during Delta and Omicron variant surges in St. Petersburg, Russia: a test-negative case–control study
Source: Respir Res. 2022 Oct 10;23:276. doi: 10.1186/s12931-022-02206-3 (PMC9549449; doi:10.1186/s12931-022-02206-3)
Supplement: Supplementary file 1 — Additional file 1: Table A1. Characteristics of all patients referred to the LDCT triage. Table A2. Effectiveness of vaccination against any and severe lung injury (sensitivity analysis performed using data from 46008patients referred to the LDCT triage. [file 12931_2022_2206_MOESM1_ESM.pdf]

Supplementary materials

Gam-COVID-Vac, EpiVacCorona, and CoviVac effectiveness against lung injury during Delta and Omicron variant surges in St. Petersburg, Russia: a test-negative case-control study

Anton Barchuk, Anna Bulina, Mikhail Cherkashin, Natalia Berezina, Tatyana Rakova, Darya Kuplevatskaya, Dmitriy Skougarevskiy, Artemiy Okhotin.

Table A1. Characteristics of all patients referred to the LDCT triage.

		Overall	No lung injury	Any lung injury	Severe lung injury
		46008	23667	22341	618
Age (mean (SD))		51.89 (16.60)	48.07 (16.55)	55.93 (15.68)	61.86 (14.48)
Age (categories (%))	18-30	4983 (10.8)	3762 (15.9)	1221 (5.5)	9 (1.5)
	31-40	8367 (18.2)	5178 (21.9)	3189 (14.3)	9 (1.5)
	41-50	8244 (17.9)	4455 (18.8)	3789 (17.0)	93 (15.0)
	51-60	8889 (19.3)	4214 (17.8)	4675 (20.9)	136 (22.0)
	60+	15525 (33.7)	6058 (25.6)	9467 (42.4)	339 (54.9)
Sex (%)	Female	29442 (64.0)	15200 (64.2)	14242 (63.7)	345 (55.8)
	Male	16566 (36.0)	8467 (35.8)	8099 (36.3)	273 (44.2)
Vaccination status	Non-vaccinated	24514 (53.3)	10686 (45.2)	13828 (61.9)	469 (75.9)
	One-dose Gam-COVID-Vac (Sputnik Light)	839 (1.8)	586 (2.5)	253 (1.1)	1 (0.2)
	Two-dose Gam-COVID-Vac (Sputnik V)	12779 (27.8)	7627 (32.2)	5152 (23.1)	95 (15.4)
	Three-dose Gam-COVID-Vac (booster)	3881 (8.4)	2562 (10.8)	1319 (5.9)	21 (3.4)
	EpiVacCorona	536 (1.2)	204 (0.9)	332 (1.5)	11 (1.8)
	CoviVac	1416 (3.1)	786 (3.3)	630 (2.8)	8 (1.3)
	No vaccination date	724 (1.6)	488 (2.1)	236 (1.1)	3 (0.5)
	Vaccine name is not reported	847 (1.8)	477 (2.0)	370 (1.7)	5 (0.8)
	Partially vaccinated	381 (0.8)	199 (0.8)	182 (0.8)	5 (0.8)
	Other vaccines or combination of vaccines	91 (0.2)	52 (0.2)	39 (0.2)	0 (0.0)
Period	Delta surge (October 1, 2021— January 9, 2022)	25813 (56.1)	10890 (46.0)	14923 (66.8)	435 (70.4)
	Omicron surge (January 10, 2022 —- April 28, 2022)	20195 (43.9)	12777 (54.0)	7418 (33.2)	183 (29.6)
Triage centre (%)	1	28433 (61.8)	15039 (63.5)	13394 (60.0)	368 (59.5)
	2	17575 (38.2)	8628 (36.5)	8947 (40.0)	250 (40.5)

Table A2. Effectiveness of vaccination against any and severe lung injury (sensitivity analysis performed using data from 46008 patients referred to the LDCT triage

	Any lung injury		Severe lung injury	
	Crude VE (95% confidence interval)	VE adjusted for age, sex and triage center (95% confidence interval)	Crude VE (95% confidence interval)	VE adjusted for age, sex and triage center (95% confidence interval)
One-dose Gam-COVID-Vac (Sputnik Light)	67% (61 to 71)	68% (62 to 72)	96% (72 to 99)	96% (74 to 99)
Two-dose Gam-COVID-Vac (Sputnik V)	48% (45 to 50)	50% (48 to 52)	72% (65 to 77)	72% (65 to 78)
Three-dose Gam-COVID-Vac (booster)	60% (57 to 63)	64% (62 to 67)	81% (71 to 88)	84% (76 to 90)
EpiVacCorona	-26% (-50 to -5)	-13% (-36 to 6)	-23% (-127 to 33)	6% (-76 to 50)
CoviVac	38% (31 to 44)	33% (25 to 40)	77% (53 to 89)	71% (42 to 86)
